# Comparison of Suicide Rates Among US Veteran and Nonveteran Populations

**DOI:** 10.1001/jamanetworkopen.2023.24191

**Published:** 2023-07-18

**Authors:** Andrew R. Morral, Terry L. Schell, Rosanna Smart

**Affiliations:** 1 RAND Corporation, Arlington, Virginia

## Abstract

This quality improvement study uses data from the US Department of Veterans Affairs to compare the relative risk of suicide among US veteran and nonveteran populations.

## Introduction

The US Department of Veterans Affairs (VA) estimates that between 2017 and 2020 veteran suicide rates were 1.57 to 1.66 times greater than nonveterans in the US, after adjusting for age and sex differences.^1^ This finding does not mean that veteran suicide rates are 1.57 to 1.66 times greater than nonveterans with the same age and sex distributions. The US government approach to age and sex adjustment compares suicide rates under counterfactual conditions in which veteran and nonveteran populations share a common age and sex distribution, namely that of the population of the US in 2000. In this study we compare the relative risk of suicide among veterans and nonveterans using the government’s method called *direct standardization* with an alternative approach that compares the true veteran population with an age- and sex-matched nonveteran population (*indirect standardization*).

## Methods

We aggregated and used 4 years (2017-2020) of data published by the VA^1^ on suicide counts and population sizes for veterans and nonveterans to calculate their relative risk of suicide using 2 standard procedures. First, we compared suicide rates using a direct standardization approach, which weights mortality rates for each demographic stratum of veteran and nonveteran populations to match the US population age and sex distribution. Second, we compared these rates using indirect standardization, which weights nonveteran suicide rates to match the age and sex distribution of the veteran population. The study was determined to be not human participant research by the RAND institutional review board. As such, it was exempt from the requirement to obtain informed consent. This study follows SQUIRE 2.0 guidelines for quality improvement reporting excellence. Analyses were conducted using Excel version 16.67 (Microsoft Corp).

## Results

The veteran population is older and includes a much higher percentage of men than does the nonveteran population (Table). These differences, and differences in the relative risk of suicide within these strata, lead to notable differences in estimates produced by the 2 methods. Although both methods show veteran risk as elevated relative to nonveterans, the direct method produces an adjusted mortality ratio of 1.59, nearly 6 times greater elevation in risk than found by indirect standardization, which produces a standardized mortality ratio of 1.10.

**Table.** Veteran and Nonveteran Suicide Rates By Demographic Strata, 2017-2020^a^

Sex and age group, y	Population in 100 000s		Suicides, No.		Suicide rate		MR^b^	Weight	
	Veteran	Nonveteran	Veteran	Nonveteran	Veteran	Nonveteran		Direct^c^	Indirect^d^
**Male**									
18-34	61.3	1488.8	3172	39 185	51.7	26.3	1.97	0.15	0.08
35-54	173.1	1468.5	6240	40 576	36	27.6	1.30	0.16	0.22
55-74	310.1	1084.9	9491	30 075	30.6	27.7	1.10	0.14	0.39
≥75	175.0	192.4	5817	8881	33.2	46.2	0.72	0.04	0.22
**Female**									
18-34	14.1	1478.5	292	9648	20.7	6.5	3.18	0.15	0.02
35-54	32.5	1634.2	555	14 491	17.1	8.9	1.92	0.16	0.04
≥55	32.3	2022.7	501	13 135	15.5	6.5	2.38	0.20	0.04

Abbreviation: MR, mortality ratio.

^a^ Data adapted from the US Department of Veterans Affairs.^1^ Counts are 4-year totals; populations are counts of person-years. Suicide mortality rates are expressed per 100 000 person-years. The estimates are population statistics not subject to sampling variance.

^b^ The unadjusted MR for veterans relative to nonveterans is 1.96, computed by summing unweighted counts over strata.

^c^ Direct weights represent the proportion of the total US population in each demographic stratum. The adjusted relative risk is 1.59 when summing over strata using direct standardization weights.

^d^ Indirect weights reflect the proportion of the veteran population in each stratum. The adjusted relative risk is 1.10 when summing over strata using indirect standardization weights.

The 2 approaches apply weights to create aggregate mortality ratios across strata (Table). Direct standardization gives far more weight to the relatively small groups of young and female veterans compared with indirect standardization, precisely the groups for which veteran suicide risk is highest relative to nonveterans.

## Discussion

Both direct and indirect standardization are legitimate methods of producing adjusted comparisons of mortality rates. Direct standardization is especially useful for comparing disease burdens across several groups, but the absolute value of such weighted rates “have no intrinsic meaning,”^2^ and do not correspond to the actual mortality risk of either of the populations being compared.^2^ When a group is demographically dissimilar from the reference population, as are veterans compared with the US population, direct estimates are strongly weighted toward the relative mortality risk of groups least well represented in the reference population used for standardization.

An advantage of the indirect standardization approach is that it counts all veteran suicides as equal in establishing relative risks. In contrast, the direct standardization approach we used would require more than 50 additional suicides among male veterans aged more than 75 years to move the resulting adjusted mortality ratio as much as a single additional suicide by a female veteran less than age 34 years.

Due to the large differences in relative suicide risk across strata, age- and sex-specific mortality ratios are the most useful statistics for most purposes. When an aggregate statistic is required to compare suicide risk among veterans and nonveterans, the correct interpretation of indirect standardization results might be more easily conveyed to general audiences because it compares actual veteran suicide rates with those of similar nonveterans.

## Notes

Supplement.
Data Sharing Statement
