# Supplementary material for: Comparison of Suicide Rates Among US Veteran and Nonveteran Populations
Source: JAMA Netw Open. 2023 Jul 18;6(7):e2324191. doi: 10.1001/jamanetworkopen.2023.24191 (PMC10354669; doi:10.1001/jamanetworkopen.2023.24191)
Supplement: Supplement. — Data Sharing Statement [file jamanetwopen-e2324191-s001.pdf]

Data Sharing Statement

Morral. Comparison of Suicide Rates Among US Veteran and Nonveteran Populations. *JAMA Netw Open*. Published July 18, 2023. doi:10.1001/jamanetworkopen.2023.24191

Data
Data available: Yes
Data types: Other (please specify)
Additional Information: Data for this study are publicly available from the Veterans Affairs Administration
How to access data: Data are available at https://www.mentalhealth.va.gov/suicide_prevention/data.asp
When available: beginning date: 01-01-2021

Supporting Documents
Document types: None

Additional Information
Who can access the data: The data are publicly available
Types of analyses: There are no restrictions other than those listed on the VA's website.
Mechanisms of data availability: Data are available for download on the VA's website.
Any additional restrictions: None
